# Blast-Induced Neurotrauma Results in Spatially Distinct Gray Matter Alteration Alongside Hormonal Alteration: A Preliminary Investigation

**DOI:** 10.3390/ijms24076797

**Published:** 2023-04-05

**Authors:** Sarah C. Hellewell, Douglas A. Granger, Ibolja Cernak

**Affiliations:** 1 Curtin Health Innovation Research Institute, Faculty of Health Sciences, Curtin University, Perth, WA 6102, Australia; 2 The Perron Institute for Neurological and Translational Science, Perth, WA 6009, Australia; 3 Institute for Interdisciplinary Salivary Bioscience Research, University of California at Irvine, Irvine, CA 92697, USA; 4 Department of Pediatrics, Johns Hopkins University School of Medicine, Baltimore, MD 21205, USA; 5 Department of Biomedical Sciences, Mercer University School of Medicine, Columbus, GA 31902, USA

**Keywords:** blast (explosion) wave-induced neurotrauma, traumatic brain injury (craniocerebral trauma), brain volume changes, occupational stress, testosterone, military

## Abstract

Blast-induced neurotrauma (BINT) frequently occurs during military training and deployment and has been linked to long-term neuropsychological and neurocognitive changes, and changes in brain structure. As military personnel experience frequent exposures to stress, BINT may negatively influence stress coping abilities. This study aimed to determine the effects of BINT on gray matter volume and hormonal alteration. Participants were Canadian Armed Forces personnel and veterans with a history of BINT (*n* = 12), and first responder controls (*n* = 8), recruited due to their characteristic occupational stress professions. Whole saliva was collected via passive drool on the morning of testing and analyzed for testosterone (pg/mL), cortisol (μg/dL), and testosterone/cortisol (T/C) ratio. Voxel-based morphometry was performed to compare gray matter (GM) volume, alongside measurement of cortical thickness and subcortical volumes. Saliva analyses revealed distinct alterations following BINT, with significantly elevated testosterone and T/C ratio. Widespread and largely symmetric loci of reduced GM were found specific to BINT, particularly in the temporal gyrus, precuneus, and thalamus. These findings suggest that BINT affects hypothalamic–pituitary–adrenal and –gonadal axis function, and causes anatomically-specific GM loss, which were not observed in a comparator group with similar occupational stressors. These findings support BINT as a unique injury with distinct structural and endocrine consequences.

## 1. Introduction

Blast-induced neurotrauma (BINT) is a common but poorly understood source of traumatic brain injury (TBI) in military personnel [1,2,3]. BINT has been described as the “signature injury” of the wars in Iraq and Afghanistan [4,5] whereby BINT accounted for an estimated 14% of deployment-related TBIs in American military personnel [6]. The cognitive, affective and neurological consequences of BINT have been well described, and include poor memory and attention, emotional processing difficulties, depression, post-traumatic stress disorder, motor deficits and hormonal alteration [7,8,9]. These symptoms are experienced acutely, and in approximately 10–15% of cases, they persist over months and years [10]. Indeed, a recent longitudinal study [11] examining participants 10 years after initial assessment [8] found that those with a history of BINT exposure had a significantly worse overall disability score compared to those not exposed to BINT [11]. The neuropathological sequelae of BINT have received less attention, with few studies conducted on gray matter (GM) volume alteration after BINT [12].

Structural MRI findings conducted to date in active military personnel, veterans and breachers exposed to BINT have reported specific cortical volume and thickness alterations in the superior frontal, middle frontal and orbitofrontal cortices [13,14,15,16,17,18,19], either bilaterally [13,14,15,19] or restricted to the left hemisphere [13,17,18,20]. Alterations to the temporal lobe have also been reported, with involvement of the superior [17] and inferior temporal gyri [21]. Conversely, several studies have reported increases in cortical thickness and volume, including in breachers with repetitive low-level injuries [22], and in veterans with comorbid chronic pain [23] and post-traumatic stress disorder [24]. Several studies have also found volumetric alterations of the thalamus, amygdala and hippocampus, key structures of the limbic system [16,25,26]. While the pathogenesis of BINT has not been fully elucidated, insights into mechanisms potentially mediating volume alteration have come from preclinical models. Primary blast overpressure affects the brain both directly through the skull and via transfer of kinetic energy from the body [2]. This may result in immediate shear stress, with downstream consequences of altered myelin ultrastructure and mitochondrial dysfunction [27], Glial activation [28], tau hyperphosphorylation [29,30] and hormonal disturbance.

Military personnel also frequently experience prolonged periods of occupational stress in both training and theatre [31,32,33], with military members significantly more likely to experience occupational stress than civilians [34]. The biological stress response centers on activation of the sympathetic–adrenal–medullar (SAM) and hypothalamic–pituitary–adrenal (HPA) axes [35,36]. While considered the major hormonal marker of stress, cortisol levels are also susceptible to environment and trauma, with a psychogenic basis for hyper- and hypocortisolism [37]. First responders and others engaged in occupations characterized by high workplace stress demonstrate significantly elevated cortisol levels related to perceptions of stress [38,39]. Testosterone levels are also inversely related to occupational stress [40,41] with concentrations decreasing as stress increases. This is also true for periods of acute enhanced stress in military personnel [42], for whom testosterone levels have been demonstrated to decrease as stress intensity rises. Similarly to BINT, occupational stress has downstream effects on working memory [43], high rates of mental ill health [44], and emotional dysregulation [45]. These alterations might adversely affect an individual’s capacity for recovery, stress coping and reduce resilience toward psychological and/or physical injuries.

Occupational stress may itself affect brain volumes. Military personnel and firefighters under high occupational stress compared to low stress counterparts manifested stress-related GM reductions in the right medial prefrontal and orbitofrontal cortices [46], left amygdala and bilateral insula [47]. Stress-related GM reductions have also been observed in the anterior cingulate and the dorsolateral prefrontal cortex [48] as well as the caudate and putamen [46,48]. Mechanistically, this may be mediated by elevated cortisol concentrations, with several of these regions susceptible to GM loss in hypercortisolism [49].

Heightened stress prior to, during and after BINT exposure may worsen the clinical manifestation of BINT. In a rodent study, the combination of chronic stress and BINT resulted in prolonged cognitive and anxiety deficits, above those seen in stress alone [50]. The combination of BINT and stress also resulted in molecular and cellular pathology, including neuronal and glial cell loss and inflammation. Mounting evidence suggests that BINT may also independently alter pituitary hormonal production [51].

Given that BINT and occupational stress have complex and overlapping effects on the brain and peripheral body systems, we sought to investigate structural brain alterations and neuroendocrine and immune system changes in BINT relative to a control group who experience similar workplace stressors. By directly comparing effects of BINT in military personnel to first responders without a history of BINT, in this study, we aimed to elucidate the effects BINT may have in people experiencing injury in the context of stressful work from those of heightened stress without injury.

## 2. Results

Participant characteristics are presented in Table 1. No significant differences were found between BINT and control participant groups with regards to age, sex and years of education. The median self-reported time since last blast exposure was 6 years, with a range of 8 months to 35 years. The median number of blast exposures was 3.5, with all participants in the BINT group experiencing at least two blast exposures in their lifetime.

### 2.1. Saliva Markers

**Figure 1:**
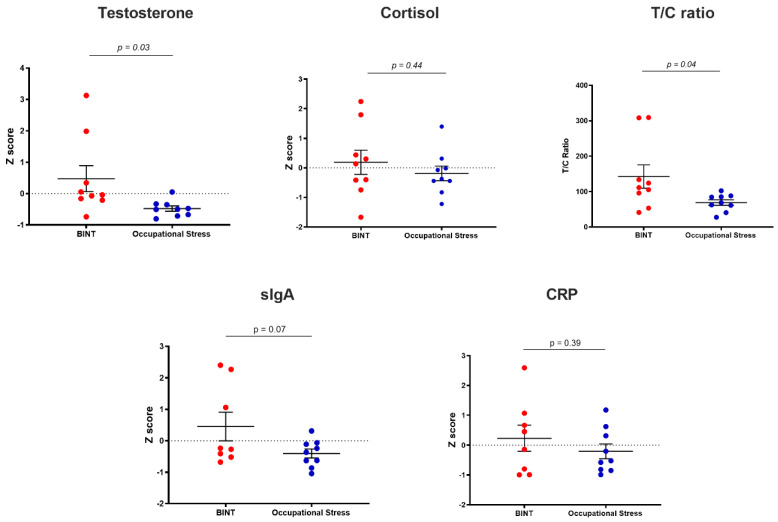
Saliva marker profiles of testosterone, cortisol, testosterone/cortisol (T/C) ratio, soluble immunoglobulin A (SIgA), C-reactive protein (CRP). Data are expressed as mean Z score plus or minus the standard error of the mean (SEM).

Saliva measurements from BINT and control groups is shown in Figure 1, with data presented as Z scores. Saliva testosterone concentration differed significantly between groups (*p* = 0.03), with the BINT group having a significantly higher mean Z score of 0.48 (±1.24) compared to controls, which had a mean Z score of −0.48 (±0.25). There were no significant differences in testosterone concentration between male and female participants, and no significant effect of age. Cortisol concentrations were not significantly different between the BINT and control groups. The ratio of testosterone and cortisol (T/C ratio) was significantly elevated in the BINT group (*p* = 0.04). No significant group differences were found for saliva markers of immunity (soluble immunoglobulin A; sIgA) and systemic inflammation (C-reactive protein; CRP).

### 2.2. Whole-Brain Voxel-Based Morphometry (VBM)

**Figure 2:**
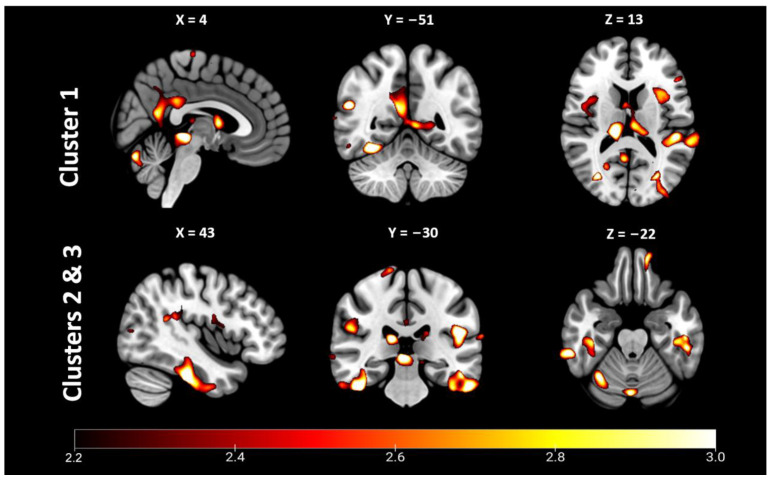
Whole-brain voxel-based morphometry revealed significant clusters of reduced gray matter in blast-induced neurotrauma (BINT) compared to occupational stress control. Statistical parametric T-score maps of depicting clusters 1–3, which were centered medially (1) and bilaterally in the temporal lobes (2 & 3). *p* < 0.05, FDR-corrected.

Whole-brain VBM findings are presented in Figure 2 and Table 2. Clusters of reduced GM were found in a regionally specific pattern throughout the brain. The largest of these was a contiguous cluster of 35,079 voxels centered medially in the right precuneus. This cluster extended anteriorly to the posterior and middle cingulate and encompassed the thalamus bilaterally. It also extended inferiorly to the cerebellar vermis, lingual gyrus bilaterally, the left calcarine gyrus and left precuneus. Clusters #2 (28,912 voxels) and #3 (15,092 voxels) were bilateral clusters of the right and left temporal lobe, respectively. Cluster #2 was centered in the right fusiform gyrus, and extended anterioinferiorly to involve the middle, inferior and superior temporal lobe, and posteriorly to encompass the cerebellar vermis, crus 1 and cerebellum lobe 6 bilaterally. Cluster #3 was centered in the left inferior temporal gyrus and extended to the fusiform gyrus. Several smaller clusters were also reflected bilaterally, such as clusters #4 (9446 voxels) and #7 (4264 voxels). These clusters involved the left (cluster #4) and right (cluster #7) superior temporal lobes, supramarginal gyrus and Rolandic operculum. In contrast, several medium-sized clusters were restricted to the left hemisphere, including cluster #5 (5981 voxels) centered on the cuneus and extending to the superior occipital gyrus and calcarine gyrus; cluster #6 (4381 voxels) which was centered in the left inferior orbitofrontal gyrus, and extended to the inferior frontal gyrus (triangular part), inferior frontal operculum, Rolandic operculum, putamen and insula; cluster #8 (3401 voxels), which was located in the middle and superior occipital gyri of the right hemisphere; cluster #9 (3369 voxels), which was centered in the right insula and extended posteriorly to the Rolandic operculum; and cluster #10, centered in the most anterior aspect of the temporal pole of the middle temporal gyrus.

### 2.3. Brain Volume and Thickness Analyses

Findings of brain volumes and cortical thickness measurements are presented in Table 3. No significant differences were found between BINT and control groups with regards to total brain volume, total gray matter, total white matter or subcortical gray matter. Likewise, no differences were found on volumetric assessment of whole subcortical structures. Examination of cortical thickness revealed significant bilateral reduction of thickness in the inferior temporal gyri (right: *p* = 0.02; left: *p* = 0.04 in BINT vs. control). Hemisphere-dependent volume alterations were found in the thalamic nuclei, with significant volumetric reduction in the left anteroventral nucleus (*p* = 0.05) and pulvinar nucleus, and right lateral (*p* = 0.02) and medial (*p* = 0.01) geniculate nuclei.

### 2.4. Correlations between Saliva Markers and Brain Volume & Thickness Measurements

The relationships between saliva markers and brain measurements were investigated by correlating significant volume and thickness findings with saliva marker mean Z scores. No statistically significant correlations were detected between any of these measures.

## 3. Discussion

In this paper, we demonstrate that BINT is associated with persistent GM volume reduction and hormonal alterations. By comparing findings in our BINT participant group to first responder peers, we were able to provide preliminary evidence that the structural and physiological effects of BINT differ to those that may be experienced as a result of occupational stress. Here, we chose to use first responders as a comparator group because first responders experience similar high-stress workplace environments, and could therefore serve as a more appropriate control to examine the effects of BINT. This would not be possible with comparison to a healthy control group, emphasizing the need for situationally relevant controls in brain injury research.

Imbalance of the steroid hormones testosterone and cortisol have been associated with aggression both in terms of impulsive (reactive) and proactive aggression where testosterone is dominant [52,53,54]. This so-called dual-hormone hypothesis [55] postulates that the HPA axis is a moderator of the hypothalamic–pituitary–gonadal (HPG) axis, such that reduction of cortisol may lead to an excess of testosterone, and vice versa. Studies have demonstrated alteration of the T/C ratio in favor of testosterone to be a driver of impulsivity and risk taking [56,57]; however, other studies have not found such a relationship in male military veterans [58,59]. In the present study, we found that the T/C ratio was significantly higher in the BINT group compared to the control group. This relationship was driven by elevations in testosterone, which was also significantly elevated in the BINT group. While not addressed in the present study, future longitudinal studies could examine the impact of chronically elevated or reduced testosterone in terms of physical and reproductive health and ageing, and the trajectory these may take when influenced by BINT.

In contrast to our findings of altered testosterone, we did not detect any differences between groups with regard to cortisol concentration. Elevated cortisol levels are more typically associated with prolonged activation of the HPA axis in stress [60], although more recent evidence suggests that hypercortisolism may be temporary, with eventual hypocortisolism maintained in the chronic phase [61]. This phenomenon may go some way to explain the cortisol data in our two participant groups, as there was large variation within each group in cortisol level. This may indicate that participants in each group experienced low, average and high levels of cortisol, respectively. We also found that saliva concentrations of sIgA and CRP followed the same directionality in the Z score change in each group (higher or positive mean Z score in BINT, and lower or negative mean Z score in occupational stress); however, there were no significant changes detected between groups for either marker. We have previously demonstrated deployment- and post-deployment-related elevations in sIgA and CRP [62], indicating that immune system activation may accompany acute and chronic stress [63,64]; however, this was not found to be the case in this participant cohort.

We also did not detect any relationships between saliva markers and brain volume or thickness alterations. As our preliminary study population was modest in size and hormones were assessed at a single timepoint, this finding is not entirely surprising. Further examination into these relationships is warranted in a larger cohort with saliva samples measured at multiple timepoints. This would allow estimation of more stable individual hormonal differences, and to determine whether brain structural alteration truly occurs independent of hormonal alteration or whether there are additive or synergistic effects.

Our VBM analyses revealed that reduced GM volumes were widespread in the BINT group compared to control, implying that BINT results in GM alteration beyond that induced by occupational stress. The largest contiguous cluster of GM alteration was found medially, extending from the precuneus to the lingual gyrus, cingulate, subcortical thalamus and cerebellum. These structures hold particular significance for a number of cognitive and physiological processes. The precuneus is highly involved in visuospatial attention visual orientation [65], episodic memory retrieval [66,67] and awareness and self-processing [68]. The precuneus has also been suggested as an important connecting hub of the default mode network [68,69] for which impaired connectivity may be related to attention deficits [70]. Volumetric reductions of the precuneus have been demonstrated in chronic (non-blast) mTBI, with the authors suggesting that it may be specifically vulnerable to long-term structural alteration [71]. The cuneus and lingual gyrus are also likewise important in inhibitory control [72,73]. This cluster also encompassed the cingulate gyrus and thalamus of the limbic system, reductions of which may have implications for cognition and emotion [74]. The cingulate is involved in diverse cognitive functions including attention, memory and emotional modulation [75,76,77]. The thalamus has a key role for integrating sensory and motor information and may be an underappreciated site of brain injury [78]. Volumetric alterations of the thalamus have been demonstrated in moderate–severe TBI [79], with the thalamus also recently highlighted as an important site of pathology in preclinical BINT models [28,80]. Due to these findings, we performed exploratory volumetric analyses of the thalamic nuclei and found significant reductions in the anteroventral nucleus, lateral and medial geniculate nuclei, and pulvinar nucleus. Given the roles of these nuclei in attention [81] auditory and visual processing [82,83], future studies could examine volumetric alterations of these nuclei and their relationships to cognition, visual and auditory dysfunction after BINT.

Tate and colleagues were amongst the first to observe cortical alterations after BINT, noting pronounced thinning of the superior temporal, superior frontal and lateral orbitofrontal gyri [17]. These changes included Heschl’s gyrus, and were found to be associated with language difficulties. Notably, speech disorders have been highlighted as an under-studied consequence of BINT [84]. While small, we also detected a cluster of GM reduction in Heschl’s gyrus, providing support for this prior work. Our VBM findings of reduced GM volume in the superior, inferior and temporal gyri add to the literature demonstrating temporal alterations after BINT [17,21]. The exploratory analyses we performed demonstrated reduction in thickness of the inferior temporal gyrus, providing additional evidence of temporal vulnerability. In agreement with the majority of the literature examining GM volume after BINT [13,14,15,16,17,18,19], we also demonstrated cortical alterations in the inferior and superior frontal gyri, confirming the vulnerability of the frontal cortex in exposure to blast.

The findings of this study should be interpreted in the context of several limitations. Our study included a small sample size, so we may have been under-powered to detect differences, particularly for the volume of subcortical brain structures. We did not conduct assessments to characterize the degree of occupational stress in our participants; however, this would be an important consideration for future studies wishing to extricate the effects of BINT from those of stress. We collected information about prior blast exposure using self-report measures regarding number of blast exposures and time since last blast exposure. While unavoidable in collecting retrospective information, self-report is prone to difficulties with recall, particularly where significant time had passed. Where many blast exposures have been experienced, there may also be difficulty remembering the circumstances and specifics of each blast. Future studies could consider longitudinal designs examining service members more frequently during training and in theatre to gain optimal information about blast exposure.

Participants in our study had a median of 3.5 blast exposures each, with all participants having at least two blast exposures in their lifetime. We were not able to determine whether these findings could be extrapolated to cohorts with fewer or greater blast exposures; however, the effect of exposure frequency is a worthwhile topic of future investigation. Finally, the median time since last blast exposure in our study was 6 years, with a range of 8 months to 35 years. This range is substantial, and there may be differences between those who have had more recent BINT and those who are years (or decades) post injury. Interestingly, Tomaiuolo and colleagues [85] examined participants with severe traumatic brain injury at one and nine years post-injury and found that GM volume remained stable over time, while white matter volumes decreased. This suggests that GM alteration may stabilize after a period of time. Due to our small sample size, we were not able to examine differences between more recent and chronic blast exposures, but this would be worthy line of investigation in future large-scale studies, as there may be differential effects of BINT in the months and years following injury. Future research also ought to determine whether there are progressive changes in volume alteration over decades, and how these might intersect with brain ageing.

## 4. Materials and Methods

### 4.1. Participants

Recruitment occurred by word-of-mouth and pre-existing relationships of researchers to military, veteran and first responder groups. Participants recruited to the BINT group were active Canadian Armed Forces (CAF) personnel and CAF veterans with at least one self-identified exposure to BINT at minimum 6 months prior to examination (*n* = 12; 10 males, 2 females; aged 40.25 ± 8.61 years. The control group consisted of emergency first responders (firefighters, paramedics, corrections officers) who experienced similarly demanding and characteristically high-stress workplace environments but had not been exposed to BINT or any other TBI (*n* = 8; 5 males, 3 females; aged 42.38 (±9.05 years).

### 4.2. Historical BINT Exposure Assessment

BINT exposure was examined using the Quantification of Cumulative Blast Exposure (QCuBE) instrument [86], for which participants documented number of BINT exposures, lifetime history of any TBI, and detailed reports of most severe BINT experiences, including body position, distance from blast, loss of consciousness and acute symptoms experienced.

### 4.3. Saliva Collection and Analysis

Whole saliva samples were collected at approximately 10 a.m. on the morning of assessment. Participants were instructed to fast for a minimum of two hours prior to arriving for testing and abstain from all beverages aside from water. Prior to sample collection, participants were instructed to rinse their mouths with water and wait 10 min to replenish saliva before commencing passive drool collection [87]. Using saliva collection aides (Salimetrics LLC, Carlsbad, CA, USA), participants filled three cryovials with approximately 2 mL of saliva each and recorded the time taken for sample collection. Saliva samples were stored at −80 °C until assay. Saliva analysis was performed at the Interdisciplinary Institute for Salivary Bioscience Research at the University of California, Irvine. Enzyme-linked immunosorbent assay (ELISA) kits (Salimetrics) were used to determine saliva concentrations of cortisol (sensitivity: 0.007 μg/dL, interassay coefficient of variation (CV) of 3.00%), testosterone (sensitivity: 1 1.00 pg/mL, interassay CV of 5.60%), testosterone/cortisol ratio (calculated as T/C × 100), sIgA (sensitivity: 2.50 μg/mL, interassay CV of 8.6%) and CRP (sensitivity: 10.00 pg/mL, interassay CV of 3.70%). Raw concentrations were converted to Z scores for analysis.

### 4.4. MRI Acquisition

MRI data were acquired using a 3 Tesla Siemens Prisma scanner (Siemens Healthcare GmbH, Erlangen, Germany) with a standard birdcage 8-channel head coil. Structural T1 weighted anatomical volumes were obtained with an AC-PC aligned SPGR sequence (axial orientation, TR = 2080 ms, TE = 4.38 ms, flip angle = 11 degrees, FOV = 256 mm, slice thickness =1 mm, voxel dimensions = 1 mm isotropic), with an acquisition time of six min.

### 4.5. Whole-Brain Voxel-Based Morphometry

T1-weighted image data were preprocessed using the Computational Anatomy Toolbox (CAT12; http://dbm.neuro.unijena.de/cat, accessed on 13 September 2019), an extension to the SPM12 software package (http://www.fil.ion.ucl.ac.uk/spm/software/spm12, accessed on 13 September 2019). Images were normalized using an affine followed by non-linear registration, corrected for bias field inhomogeneities, and registered to the standard MNI space using high-dimensional DARTEL normalization. Images were then segmented into gray matter, white matter and cerebrospinal fluid components, accounting for partial volume effects and applying a hidden Markov random field model, which incorporates spatial prior information of adjacent voxels into the segmentation estimation. The warped tissue type images were modulated to preserve the volume of a particular tissue within a voxel by multiplying voxel values in the segmented images by the Jacobian determinants derived from the spatial normalization step. Finally, images were smoothed with a full-width half-maximum kernel of 8 mm. Brain regions were labeled and examined with reference to the Automated Anatomical Labeling (AAL) atlas.

### 4.6. Exploratory Subcortical Volume and Cortical Thickness Analysis

Based on the prior research findings of decreased limbic structural volume and reduced thickness of the frontal and temporal cortices after BINT, we performed further exploratory analyses to determine whether these alterations were also present in our cohort. Cortical reconstruction and volumetric segmentation were performed using the FreeSurfer Recon-All pipeline (v. 6.0) (http://surfer.nmr.mgh.harvard.edu/, accessed on 13 September 2019). The full methods and procedures are described elsewhere [88,89]. Briefly, processing includes motion correction and averaging, removal of non-brain tissue and automated Talairach transformation, followed by segmentation of gray and white matter, subcortical structures, and parcellation of the cerebral cortex. The thalamus was then further segmented into 25 individual thalamic nuclei using FreeSurfer’s thalamic parcellation pipeline [90], which uses a Bayesian segmentation method based on a probabilistic atlas derived from histology.

### 4.7. Statistical Analysis

Raw saliva values were converted to Z scores and expressed graphically as mean Z score ± standard deviation (SD). Brain parcellation measures were expressed relative to total brain volume (total gray matter + total white matter) to account for differences in head size. Brain volumetric, thickness and saliva markers were examined for normality using the D’Agostino–Pearson omnibus K2 test, and differences were evaluated using two-tailed independent sample *t*-tests to compare differences between BINT and control groups. Analyses were performed at the voxel level (comparisons at every voxel) for VBM data, with an uncorrected voxel threshold of *p* ≤ 0.001. Data were then reported where clusters had a minimum size of 100 voxels, with a cluster extent threshold of *p* ≤ 0.05 corrected for false discovery rate. Correlations between brain volume/thickness and saliva markers were calculated using Pearson’s correlation coefficient, after confirmation of normal distribution. GraphPad Prism software (v8.2) was used for all analyses (GraphPad, La Jolla, CA, USA). Statistical significance was considered where *p* ≤ 0.05.

## 5. Conclusions

Gray matter volume loss and hormonal alteration occur in military personnel with a history of BINT. These findings support BINT as a unique clinical injury causing discrete and distinct structural and endocrine changes and provide additional preliminary evidence suggesting the effects of BINT may be distinct from those of occupational stress.

## Figures and Tables

**Table 1:** Participant and demographic characteristics.

Variable	BINT (*n* = 12)	Control (*n* = 8)	*p*-Value
Age (mean, standard deviation)	40.25 (±8.61)	42.38 (±9.05)	0.60
Sex (number M/F)	10/2	5/3	0.35
Education (years, standard deviation)	14.18 (±1.68)	15.13 (±0.83)	0.12
Time since last blast exposure (median years, range)	6 (0.66–35)	-	-
Number of blast exposures (median, range)	3.5 (2–5000)	-	-

**Table 2:** Top 15 VBM clusters in which gray matter volume was significantly reduced in BINT participants compared to occupational stress control participants.

Cluster Number	Hemisphere	Peak Structure	Peak Coordinates			Cluster Size (Voxels)	Z Score	FDR-Corrected *p*-Value
			x	y	z			
1	Right	Precuneus	6	−53	18	35,079	4.8	*p* < 0.001
2	Right	Fusiform gyrus	36	−63	−16	28,912	5	*p* < 0.001
3	Left	Inferior temporal gyrus	−34	−19	−39	15,094	4.7	*p* < 0.001
4	Left	SupraMarginal gyrus	−48	−30	20	9446	4	*p* < 0.001
5	Left	Cuneus	−2.0	−89	34	5981	4.2	*p* < 0.001
6	Left	Inferior frontal gyrus, orbitofrontal part	−43	29	−3	4381	4	*p* < 0.01
7	Right	Middle temporal gyrus	53	50	21	4264	4.6	*p* < 0.001
8	Right	Middle occipital gyrus	33	−72	17	3401	4.9	*p* < 0.001
9	Right	Insula	39	−5	23	3369	2.5	*p* < 0.05
10	Left	Temporal pole, middle temporal gyrus	−54	13	−33	2520	3.1	*p* < 0.01
11	Left	Superior frontal gyrus, orbital part	−11	39	−28	2064	3.5	*p* < 0.05
12	Left	Supplementary motor area	−7	20	51	2054	2.9	*p* < 0.05
13	Left	Inferior frontal gyrus, triangular part	−53	29	20	1526	2.8	*p* < 0.05
14	Right	Precentral gyrus	22	−26	72	1520	2.8	*p* < 0.05
15	Left	Pallidum	−11	0	0	841	2.9	*p* < 0.05

**Table 3:** Volumetric and cortical thickness analyses.

Brain Structure	BINT (Mean ± SD)	Stress (Mean ± SD)	*p*-Value
Total Brain Volume	1156 (±111)	1140 (±112)	0.77
Total Gray Matter	557 (±25)	547 (±21)	0.49
Total White Matter	449 (±26)	453 (±21)	0.75
Subcortical gray matter	5.17 (±0.19)	5.15 (±0.19)	0.87
Inferior temporal gyrus, right	2.58 (±0.09)	2.72 (±0.15)	0.02
Inferior temporal gyrus, left	2.64 (±0.09)	2.77 (±0.13)	0.04
Anteroventral thalamic nucleus, right	159.90 (±25.60)	181.70 (±24.17)	0.07
Anteroventral thalamic nucleus, left	126.40 (±21.23)	149.50 (±18.12)	0.05
Lateral geniculate nucleus, right	277.80 (±30.02)	314.00 (±27.62)	0.02
Lateral geniculate nucleus, left	284.60 (±33.84)	287.10 (±27.37)	0.89
Medial geniculate nucleus, right	105.40 (±19.83)	129.60 (±10.85)	0.01
Medial geniculate nucleus, left	102.60 (±20.10)	116.70 (14.08)	0.18
Pulvinar nucleus, right	290.10 (±36.47)	316.50 (±13.32)	0.18
Pulvinar nucleus, left	260.90 (±45.04)	308.90 (±43.57)	0.05

All measures are reported in mm^2^ and adjusted relative to total brain volume.

## Data Availability

The data presented in this study may be available on reasonable request from the corresponding author.
